# Altered resting-state functional connectivity of insula in children with primary nocturnal enuresis

**DOI:** 10.3389/fnins.2022.913489

**Published:** 2022-07-19

**Authors:** Shaogen Zhong, Jiayao Shen, Mengxing Wang, Yi Mao, Xiaoxia Du, Jun Ma

**Affiliations:** ^1^Department of Developmental and Behavioral Pediatrics, Shanghai Children’s Medical Center, School of Medicine, Shanghai Jiao Tong University, Shanghai, China; ^2^Department of Nephrology, Shanghai Children’s Medical Center, School of Medicine, Shanghai Jiao Tong University, Shanghai, China; ^3^College of Medical Imaging, Shanghai University of Medicine and Health Sciences, Shanghai, China; ^4^School of Psychology, Shanghai University of Sport, Shanghai, China

**Keywords:** arousal, functional connectivity, insula, prefrontal cortex, primary nocturnal enuresis

## Abstract

**Objective:**

Primary nocturnal enuresis (PNE) is a common developmental condition in school-aged children. The objective is to better understand the pathophysiology of PNE by using insula-centered resting-state functional connectivity (rsFC).

**Methods:**

We recruited 66 right-handed participants in our analysis, 33 with PNE and 33 healthy control (HC) children without enuresis matched for gender and age. Functional and structural MRI data were obtained from all the children. Seed-based rsFC was used to examine differences in insular functional connectivity between the PNE and HC groups. Correlation analyses were carried out to explore the relationship between abnormal insula-centered functional connectivity and clinical characteristics in the PNE group.

**Results:**

Compared with HC children, the children with PNE demonstrated decreased left and right insular rsFC with the right medial superior frontal gyrus (SFG). In addition, the bilateral dorsal anterior insula (dAI) seeds also indicated the reduced rsFC with right medial SFG. Furthermore, the right posterior insula (PI) seed showed the weaker rsFC with the right medial SFG, while the left PI seed displayed the weaker rsFC with the right SFG. No statistically significant correlations were detected between aberrant insular rsFC and clinical variables (e.g., micturition desire awakening, bed-wetting frequency, and bladder volume) in results without global signal regression (GSR) in the PNE group. However, before and after setting age as a covariate, significant and positive correlations between bladder volume and the rsFC of the left dAI with right medial SFG and the rsFC of the right PI with right medial SFG were found in results with GSR in the PNE group.

**Conclusion:**

To the best of our knowledge, this study explored the rsFC patterns of the insula in children with PNE for the first time. These results uncovered the abnormal rsFC of the insula with the medial prefrontal cortex without and with GSR in the PNE group, suggesting that dysconnectivity of the salience network (SN)-default mode network (DMN) may involve in the underlying pathophysiology of children with PNE. However, the inconsistent associations between bladder volume and dysconnectivity of the SN-DMN in results without and with GSR need further studies.

## Introduction

Primary nocturnal enuresis (PNE), also called bed-wetting, is a common developmental condition defined as intermittent involuntary voiding during sleep in children with a minimum of 5 years (Austin et al., 2016), with a prevalence of approximately 5–10% for 7-year-old (Nevéus et al., 2020). Children suffering from PNE tend to experience a low quality of life, poor self-esteem, and bad social relationships (Caldwell et al., 2013; Nevéus et al., 2020). Although children with PNE have a spontaneous remission rate of 15% per year, frequent enuresis without any intervention will be more likely to persist into adulthood (Nevéus et al., 2020).

Currently, arousal dysfunction, nocturnal polyuria, and abnormal bladder function are usually regarded as the three main pathophysiological factors of PNE (Caldwell et al., 2013; Pedersen et al., 2020). Although this concept of pathophysiology can be favorable for clinicians to treat each child (Pedersen et al., 2020), some children with PNE are still resistant to all the available treatments (Nevéus et al., 2020). This largely results from our little understanding of the exact pathogenesis underlying PNE. Over the past decade, neuroimaging studies have found a few brain regions that are reported to differ in PNE, including the medial prefrontal cortex (mPFC), bilateral cingulate gyri, insula, thalamus, periaqueductal gray (PAG) matter, and the cerebellum (Lei et al., 2012a,b; Wang et al., 2018, 2019; Zhang et al., 2021). In addition to the delayed development of the central nervous system, the inability to wake up in response to the desire to void is also an essential role of PNE (Pedersen et al., 2020). In clinical practice, parents always complain that their children with PNE have difficulty being aroused. Therefore, the speculation could be that long-term stimulus during the bladder filling phase results in recurrent episodes of cortical arousal, which, in turn, leads to an inability to arouse completely (Yeung et al., 2008). However, the actual reason why children have difficulty waking up from the full bladder condition remains unknown.

The neural control model of the bladder proposed by Griffiths mainly consists of the default mode network (DMN) and the salience network (SN) in humans (Griffiths, 2015). According to the working model, the mPFC is a major part of the DMN that is active at rest, but deactivated when attention is required (Raichle, 2015). As well as the well-known mPFC, the insula, an integrated component of the SN, also plays a role in maintaining continence, which is activated both normally and abnormally in various situations involving sensation (Blok et al., 1998; Walter et al., 2021). Several studies have already demonstrated structural and functional alterations in the insula of children with PNE. Lei et al. (2012c) uncovered that mean diffusivity increased in the insula and they also revealed that the bilateral cingulate gyri and insula were activated less during response inhibition in children with PNE (Lei et al., 2012a). Zheng et al. (2021) found that regional homogeneity increased in the left insula, while the fractional amplitude of low-frequency fluctuation decreased in the right insula in children with PNE. In addition to involvement of bladder sensation, the insula is also engaged in working memory (Cai et al., 2021), decision-making (Loued-Khenissi et al., 2020), interoception (Chen et al., 2021), state switching (Menon and Uddin, 2010), and emotional process (Gasquoine, 2014). Based on prior functional MRI (fMRI) studies (Zhang et al., 2015; Jiang et al., 2019; Wang et al., 2019), children with PNE also have potential impairments in working memory, attention cognition, and emotional responses.

Previous studies have divided the insula into three main subregions: the ventral anterior insula (vAI), dorsal anterior insula (dAI), and posterior insula (PI) (Deen et al., 2011; Chang et al., 2013). The aberrant functional connectivity patterns in the insular cortex have been investigated in patients with various psychiatric and neurodevelopmental disorders, such as schizophrenia (Tian et al., 2019; Sheffield et al., 2020), autism spectrum disorder (Yamada et al., 2016; Guo et al., 2019), attention-deficit/hyperactivity disorder (Zhao et al., 2017; Viering et al., 2021), and posttraumatic stress disorder (Sheynin et al., 2020; Fonzo et al., 2021). However, little is known about the functional connectivity of the insula and its subregions in children with PNE.

In this present study, we mainly used a seed-based resting-state functional connectivity (rsFC) method to explore rsFC patterns in the insula and its subregions of children with PNE. Based on prior studies, we primarily hypothesized that children with PNE would demonstrate significantly abnormal rsFC patterns in the insula and its subregions with other brain regions (especially regions in DMN) relative to HC children. We also expected significant associations between the above rsFC results and clinical variables (e.g., micturition desire awakening, bed-wetting frequency, and bladder volume) in the PNE group.

## Materials and methods

### Participants

The patients and healthy control (HC) children were, respectively, recruited from an outpatient setting in Shanghai Children’s Medical Center and by advertisement. Our present study included 33 children with PNE (17 males; aged 6–13 years) and 33 HC children (17 males; aged 6–12 years) matched for gender and age, which were all right-handed. Ethical approval was obtained from the Institutional Review Board (IRB) of Shanghai Children’s Medical Center, School of Medicine, Shanghai Jiao Tong University (No. SCMC-201014). The present study was conducted based on the Declaration of Helsinki and the ethical standards of IRB. All the guardians and their children provided written informed consent before study enrollment.

All the patients were diagnosed by senior pediatricians based on the criteria of the International Children’s Continence Society (ICCS) and had bed-wetting with one episode per month or more for at least 3 months (Austin et al., 2016). Their urinary tests were normal, without glucosuria and leukocytes. Ultrasound examination of their urinary systems uncovered no organic problems in the kidney, urinary tract, and bladder. Moreover, the bladder volume was acquired by the ultrasound when patients had a strong desire to void. We also collected the age, gender, and frequency of bed-wetting by questionnaire. The micturition desire awakening was rated by two interviewers. HC children were no enuresis since their 5 years. The clinical characteristics of all the subjects are shown in Table 1.

**TABLE 1:** Demographic and clinical data in PNE and HC children.

Baseline characteristic	PNE group (*n* = 33)	HC group (*n* = 33)	*P*-value
Age, years, median (IQR)	8 (7–10)	8 (7–10)	0.896
Gender, n (%)			1.000
Male	17 (51.5)	17 (51.5)	
Female	16 (48.5)	16 (48.5)	
Handedness, n (%)			1.000
Right	33 (100)	33 (100)	
Left	0 (0)	0 (0)	
Mean FD, mean (SD)	0.09 (0.04)	0.11 (0.06)	0.185
Bladder volume, ml, mean (SD)	174.64 (78.35)	NA	NA
Bed-wetting frequency, per week, median (IQR)	4 (1.5–6.5)	NA	NA
Micturition desire-awakening, n (%)			NA
Wake up after urinating little	3 (9.1)	NA	
Wake up after urinating more	2 (6.1)	NA	
Wake up after emptying the bladder	6 (18.2)	NA	
Inability to wake up after emptying the bladder	22 (66.7)	NA	

PNE, primary nocturnal enuresis; HC, healthy control; IQR, interquartile range; FD, framewise displacement; SD, standard deviation; NA, not applicable.

The inclusion criteria of all the participants were as follows: 5–18 years; right-handedness; with an IQ above 75 (Wechsler Intelligence Scale for Children-Revised); a clinical assessment by senior developmental and behavioral pediatricians; without a history of other illnesses causing bed-wetting (e.g., diabetes mellitus, epilepsy, and urinary infection); without any history of psychiatric or neurological diseases (e.g., intellectual disability, attention-deficit/hyperactivity disorder, autism spectrum disorder, and cerebral palsy); and without receiving any treatments or drugs about antienuresis before MRI scanning.

The exclusion criteria of all the participants were as follows: with any daytime lower urinary tract symptoms; left-handedness; with an IQ below 75; contraindications for MRI; with obvious head movement (translation > 3 mm, rotation > 3°) on these brain images; and receiving any other antipsychotics.

### Neuroimaging data acquisition

The functional and structural MRI data were acquired on the 3.0 T MR imaging system (Prisma, Siemens, Germany) at Shanghai Key Laboratory of Magnetic Resonance (East China Normal University, Shanghai, China). Sequence parameters of resting-state fMRI (rs-fMRI) were set as follows: repetition time (TR)/echo time (TE) = 2,000/30 ms, acquisition time = 486 s, volume number = 240, acquisition matrix = 64 × 64, voxel size = 3.5 mm × 3.5 mm × 3.5 mm, flip angle = 90°, field of view (FOV) = 224 mm × 224 mm, and slice number = 33. All the children were told to stay awake and still, keeping their eyes closed during scanning. We also collected high-resolution T1-weighted images from all the children; sequence parameters were set as follows: slice thickness = 1 mm, inversion time = 1,100 ms, acquisition matrix = 256 × 256, TR/TE = 2,530/2.98 ms, flip angle = 7°, FOV = 256 mm × 256 mm, voxel size = 1 mm × 1 mm × 1 mm, and 192 slices (scan time of 361 s).

### Neuroimaging data preprocessing

All the MRI data were preprocessed in MATLAB 2014a (MathWorks Incorporation, Natick, Massachusetts, United States) using RESTplus version 1.24 (Jia et al., 2019), a toolkit based on SPM12 (Wellcome Trust Centre for Neuroimaging, University College London, United Kingdom).^1^ Preprocessing included the following steps: (1) Removing the first 10 time points of each rs-fMRI data for participants’ acclimatization and signal’s stability; (2) slice timing to correct the remaining time points; (3) realignment; (4) normalizing to the Montreal Neurological Institute (MNI) template and resampling into a new 3 mm × 3 mm × 3 mm voxel size by using T1 image unified segmentation; (5) smoothing with a Gaussian kernel [full width at half maximum (FWHM) of 6 mm]; (6) detrending; (7) regressing common covariates out, including Friston’s 24 head motion parameters to reduce the confound of head motion (Friston et al., 1996), white matter (WM) and cerebrospinal fluid (CSF) signals; and (8) filtering (0.01–0.08 Hz). There were no subjects excluded for excessive distortions and head motion (translation > 3 mm, rotation > 3°). We also calculated the mean framewise displacement (FD) described by Jenkinson et al. (2002) to evaluate head motion differences between the groups (Table 1). Global signal regression (GSR) should be included during preprocessing is complex and controversial (Weissenbacher et al., 2009; Liu et al., 2017; Aquino et al., 2020). Thus, we also performed an analysis with GSR and the results are demonstrated in Supplementary Table 1 and Supplementary Figures 1–3.

### Functional connectivity analysis

Seed-based rsFC analyses were processed using the RESTplus version 1.24 toolkit. First, the left and right insula seeds, based on the Anatomical Automatic Labeling (AAL) template (Tzourio-Mazoyer et al., 2002), were determined as regions of interest (ROIs). Then, another six spherical ROIs (radius = 6 mm) were determined based on one previous research investigating insular rsFC (Deen et al., 2011), including the left (−33, 13, −7) and right (32, 10, −6) vAI, the left (−38, 6, 2), and right (35, 7, 3) dAI, and also the left (−38, −6, 5) and right (35, −11, 6) PI in MNI coordinates. The average time series of each insula seed was, respectively, calculated in each subject to generate correlation maps by voxel-wise correlation coefficients. Finally, by using Fisher’s r-to-z transformation, we converted these correlation coefficients to *z*-values to improve the normality.

### Statistical analysis

Clinical and behavioral variables were processed via the statistical package SPSS version 25.0 (IBM Incorporation, SPSS). We applied the Shapiro–Wilk test to evaluate the normality of continuous data. After that, a parametric test (i.e., two-sample *t*-test) was performed for mean FD and bladder volume and non-parametric tests (i.e., the Mann–Whitney *U*-test or the chi-squared test) were conducted for age, bed-wetting frequency, gender, etc. There were statistically significant differences when *P* < 0.05 in all the above statistical analyses. In the meanwhile, descriptive statistics were used for all the clinical and behavioral variables. Continuous data were shown as mean (SD) or median [interquartile range (IQR)], depending on the data distribution. Categorical data were expressed as frequency (percentage).

Statistical analysis of rsFC was carried out using the RESTplus version 1.24 toolkit. Group comparisons on the rsFC *z*-values derived from each insula seed between the PNE and HC groups were carried out via the two-sample *t*-tests. By using a two-tailed Gaussian Random Field (GRF) correction, we carried out multiple comparison corrections for results of the left and right insula seeds (two-tailed GRF correction, *P* < 0.01 at single voxel, and *P* < 0.05 at cluster level) and the other six insula seeds (two-tailed GRF correction, *P* < 0.005 at single voxel, and *P* < 0.05 at cluster level). Furthermore, the abnormal FC *z*-values based on each seed after GRF correction were extracted. Then, we performed Pearson’s correlation analysis between these aberrant FC *z*-values and clinical variables (e.g., bed-wetting frequency and bladder volume) in the PNE group. Spearman’s correlation analysis was conducted between these abnormal FC *z*-values and micturition desire awakening in the PNE group. To control for confounding factors, further partial correlation analysis was performed if the results of simple correlation analysis were significant. The statistically significant *P*-value was considered as < 0.05 in this study.

## Results

### Demographic and clinical characteristics

Overall, 66 right-handed participants, 33 with PNE [8 (7–10) years] and 33 HC children without enuresis [8 (7–10) years], were recruited for this study. There were no significant (*P* > 0.05) differences detected for age and gender between the groups and the other clinical characteristics of all the participants are also shown in Table 1.

### Group comparisons of resting-state functional connectivity

**FIGURE 1:**
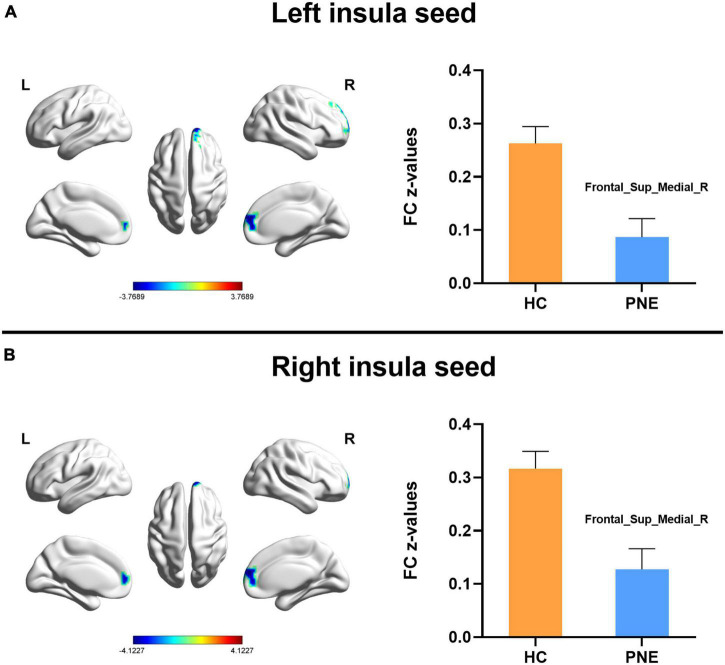
Comparisons of the left and right insula-centered functional connectivity between PNE and HC children. The **(A)** left and **(B)** right insula seeds indicated reduced functional connectivity with Frontal_Sup_Medial_R (two-tailed GRF correction, *P* < 0.01 at single voxel, and *P* < 0.05 at cluster level). PNE, primary nocturnal enuresis; HC, healthy control; Frontal_Sup_Medial, medial superior frontal gyrus; L, left; R, right. Color scales: *t*-value; Error bars: SEM.

**FIGURE 2:**
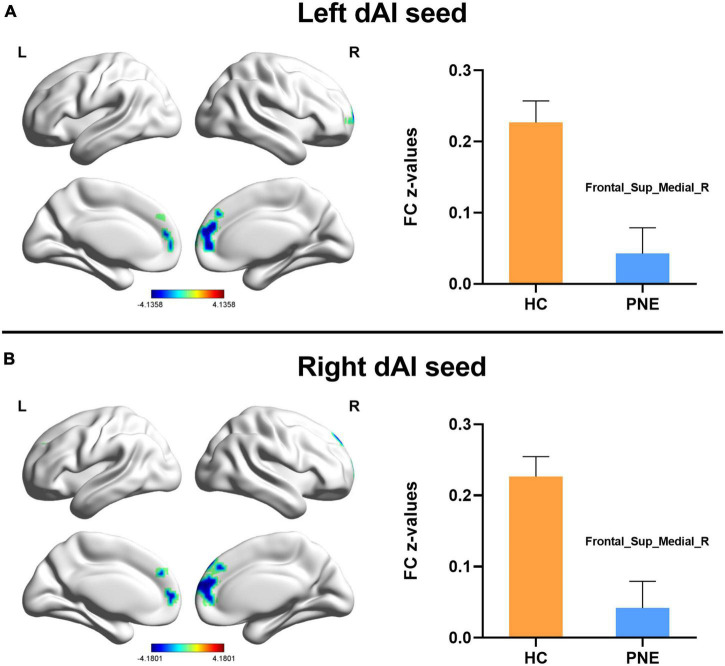
Comparisons of the dorsal anterior insula-centered functional connectivity between PNE and HC children. The **(A)** left and **(B)** right dAI seeds indicated reduced functional connectivity with Frontal_Sup_Medial_R (two-tailed GRF correction, *P* < 0.005 at single voxel, and *P* < 0.05 at cluster level). PNE, primary nocturnal enuresis; HC, healthy control; dAI, dorsal anterior insula; Frontal_Sup_Medial, medial superior frontal gyrus; L, left; R, right. Color scales: *t*-value; Error bars: SEM.

**FIGURE 3:**
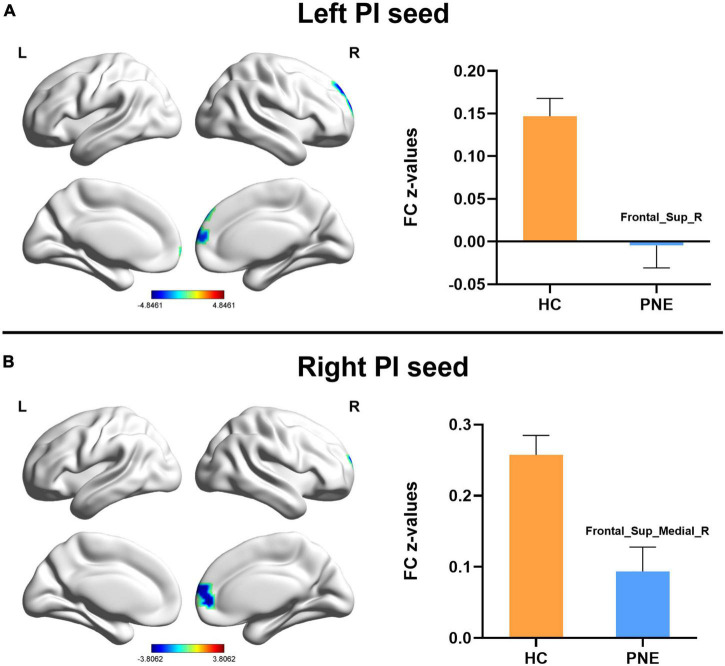
Comparisons of the posterior insula-centered functional connectivity between PNE and HC children. The **(A)** left PI seed indicated reduced functional connectivity with Frontal_Sup_R, while the **(B)** right PI seed showed decreased functional connectivity with Frontal_Sup_Medial_R (two-tailed GRF correction, *P* < 0.005 at single voxel, and *P* < 0.05 at cluster level). PNE, primary nocturnal enuresis; HC, healthy control; PI, posterior insula; Frontal_Sup_Medial, medial superior frontal gyrus; Frontal_Sup, superior frontal gyrus; L, left; R, right. Color scales: *t*-value; Error bars: SEM.

Generally, compared with HC children, the children with PNE presented weaker insula-centered rsFC (Table 2). First, the left and right insula seeds exhibited decreased rsFC with the right medial superior frontal gyrus (SFG) (two-tailed GRF correction, *P* < 0.01 at single voxel, and *P* < 0.05 at cluster level) (Figure 1). Second, the bilateral dAI seeds similarly showed reduced rsFC with right medial SFG (two-tailed GRF correction, *P* < 0.005 at single voxel, and *P* < 0.05 at cluster level) (Figure 2). Third, the right PI seed also demonstrated decreased rsFC with right medial SFG, whereas the left PI seed indicated decreased rsFC with the right SFG (two-tailed GRF correction, *P* < 0.005 at single voxel, and *P* < 0.05 at cluster level) (Figure 3). Nevertheless, there were no significant clusters based on the vAI seeds (two-tailed GRF correction, *P* < 0.005 at single voxel, and *P* < 0.05 at cluster level).

**TABLE 2:** Brain regions showing significant differences in functional connectivity with insula or its subregions between PNE and HC children.

Seed	Brain region	Cluster size	MNI coordinates			Peak *t*-value
			*X*	*Y*	*Z*	
Left insula	Frontal_Sup_Medial_R (BA 9)	305	6	54	18	–3.7689
Right insula	Frontal_Sup_Medial_R (BA 9)	324	6	54	18	–4.1227
Left dAI	Frontal_Sup_Medial_R (NA)	236	12	51	18	–4.1358
Right dAI	Frontal_Sup_Medial_R (BA 9)	375	9	57	39	–4.1801
Left PI	Frontal_Sup_R (BA 10)	268	15	66	18	–4.8461
Right PI	Frontal_Sup_Medial_R (BA 10)	203	12	66	21	–3.8062

PNE, primary nocturnal enuresis; HC, healthy control; MNI, Montreal Neurological Institute; BA, Brodmann areas; dAI, dorsal anterior insula; PI, posterior insula; Frontal_Sup_Medial_R, right medial superior frontal gyrus; Frontal_Sup_R, right superior frontal gyrus; NA, not applicable.

In results with GSR, the rsFC patterns of the insula and its subregions with other brain regions were similar to the above results without GSR. The difference in results with GSR is that there were no significant results based on the left PI seed, while the right vAI seed showed significantly decreased rsFC with right medial SFG (Supplementary Table 1 and Supplementary Figures 1–3).

### Relationship between resting-state functional connectivity and clinical characteristics

**FIGURE 4:**
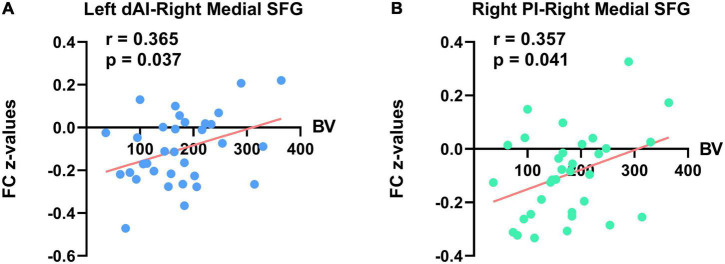
Significant correlations between bladder volume (BV) and FC *z*-values of the insula with other brain regions in PNE subjects (with global signal regression). The **(A)** left panel indicates a significant positive correlation between rsFC of left dAI with right medial SFG and BV. The **(B)** right panel indicates a significant positive correlation between rsFC of right PI with right medial SFG and BV. dAI, dorsal anterior insula; SFG, superior frontal gyrus; PI, posterior insula.

Simple correlation analysis demonstrated no statistically significant results between all the clinical variables and altered FC *z*-values of the insula with other brain regions in results without GSR in the PNE group (Supplementary Table 2). There were also no significant correlations between bed-wetting frequency or micturition desire awakening and abnormal FC *z*-values in results with GSR in the PNE group. However, in results with GSR, there were significant positive correlations between bladder volume and the rsFC of the left dAI with right medial SFG (*r* = 0.365, *P* = 0.037) and the rsFC of the right PI with right medial SFG in the PNE group (*r* = 0.357, *P* = 0.041) (Table 3 and Figure 4). Due to the effect of age on bladder volume, a further partial correlation analysis was conducted and the results remained significantly positive [(*r* = 0.360, *P* = 0.043) and (*r* = 0.354, *P* = 0.047), respectively].

**TABLE 3:** Simple correlation analysis between clinical characteristics and FC *z*-values of the insula or its subregions with other brain regions in PNE subjects (with global signal regression).

Seed	Brain region	Clinical characteristic	*r*	*P*
Left insula	Frontal_Sup_ Medial_R (BA 10)	MDA	0.171	0.340
		Frequency	0.107	0.553
		BV	0.315	0.074
Right insula	Frontal_Sup_ Medial_R (BA 10)	MDA	0.072	0.691
		Frequency	0.181	0.314
		BV	0.216	0.227
Right vAI	Frontal_Sup_ Medial_R (BA 9)	MDA	0.147	0.415
		Frequency	0.188	0.295
		BV	0.113	0.530
Left dAI	Frontal_Sup_ Medial_R (NA)	MDA	0.112	0.534
		Frequency	0.205	0.252
		BV	0.365	0.037*
Right dAI	Frontal_Sup_ Medial_R (BA 10)	MDA	0.033	0.854
		Frequency	0.188	0.294
		BV	0.213	0.233
Right PI	Frontal_Sup_ Medial_R (BA 10)	MDA	–0.093	0.608
		Frequency	–0.058	0.749
		BV	0.357	0.041*

PNE, primary nocturnal enuresis; MDA, micturition desire-awakening; Frequency, bed-wetting frequency per week; BV, bladder volume; dAI, dorsal anterior insula; PI, posterior insula; Frontal_Sup_Medial_R, right medial frontal superior gyrus; Frontal_Sup_R, right superior frontal gyrus; BA, Brodmann areas; NA, not applicable.

*P < 0.05.

## Discussion

To the best of our knowledge, this is the first study to explore the rsFC patterns of the insula and its subregions with other brain regions in children with PNE. First, compared with HC children, we mainly found that altered rsFC of the bilateral insula seeds with right medial SFG in PNE children in results without and with GSR. Second, children with PNE also displayed abnormal rsFC of the bilateral dAI with right medial SFG in results without and with GSR. Third, the right PI seed showed weaker rsFC with right medial SFG in children with PNE in results without and with GSR, while the left PI insula seed only demonstrated reduced rsFC with right SFG in results without GSR. Interestingly, the reduced rsFC of vAI with right medial SFG in the PNE group was only revealed in results with GSR. In the results without GSR, there were no significant relationships between any clinical variables and rsFC *z*-values in the PNE group. However, before and after setting age as a covariate, significant and positive correlations between bladder volume and the rsFC of the left dAI with right medial SFG, and the rsFC of the right PI with right medial SFG were found in results with GSR in the PNE group. These findings indicated that dysconnectivity of the SN-DMN may involve in the underlying pathophysiology of children with PNE.

Compared with HC children, our study reported that the bilateral insula seeds demonstrated decreased rsFC with right medial SFG in children with PNE. The right medial SFG is located in the mPFC, which is regarded as a critical node of the DMN (Franco et al., 2009). The well-known DMN performs functional connectivity enhancement and is active in the resting condition, but it is deactivated when attention is required (Griffiths, 2015). It plays a tremendous role in emotional processing (Spies et al., 2017), self-referential mental activity (Raichle, 2015), and monitoring the self and surrounding environment (Gusnard et al., 2001). Previous task fMRI studies have shown that the DMN is involved in the potential dysfunctions of emotional responses (Wang et al., 2019), response inhibition (Lei et al., 2012a), and working memory (Zhang et al., 2015) in children with PNE during the relevant tasks. In addition, abnormalities of local brain activity in mPFC have been identified in extensive studies and may be related to the cortical control mechanisms of PNE (Lei et al., 2012b; Schulz-Juergensen et al., 2013; Zhu et al., 2019). Besides abnormalities of local brain activity, the key brain regions of DMN, dorsal attention network (DAN), and ventral attention network (VAN) were damaged and likely related to attention impairment in children with PNE (Jiang et al., 2019). The simple model of the lower urinary tract (LUT) control presented by Griffiths demonstrated that the DMN and SN are two crucial brain networks to sustain continence (Griffiths, 2015). The mPFC, the parahippocampal complex, and the midbrain periaqueductal gray (PAG) are three integral components of circuits related to micturition control. Another essential circuit, including the insula and dorsal anterior cingulate cortex associated with bladder sensation (Griffiths, 2015) and interoceptive awareness (Menon and Uddin, 2010), belongs to the SN. Moreover, the reduced result in the right medial SFG mainly lies in the Brodmann area (BA) 9 (Table 2). Interestingly, BA9 is a part of dorsolateral PFC (dlPFC), which is activated by bladder filling if the SN is also strongly activated (Griffiths, 2015; Carlén, 2017). These results suggested that children with PNE had dysfunctions in the DMN and SN, which may induce emotional problems, working memory dysfunction, attention impairment, and bladder control dysfunction. The right medial SFG is also a primary area of the supplementary motor area (SMA) (Wei et al., 2021). The SMA, a part of the SN (Griffiths, 2015), is essential to motor-related functions, higher-order cognitive control (Hertrich et al., 2016), and bladder control (Griffiths, 2015). Based on these findings, we speculated that the dysconnectivity of SN-DMN might exert a special role in the pathogenesis of children with PNE.

Our further results demonstrated that the rsFC of bilateral dAI seeds with right medial SFG was also weaker in subjects with PNE. In previous rsFC studies, the dAI is related to cognitive control processing, connected with frontal, dorsal anterior cingulate cortex, and parietal areas (Deen et al., 2011; Uddin et al., 2017). Furthermore, the insula, especially the anterior insula, displayed its role in generating normal series of bladder sensations (Griffiths et al., 2007). Therefore, the aberrant rsFC between key brain regions of the SN and the DMN may lead to abnormal bladder sensations, such as the desire to void. However, the left and right vAIs, related to emotion processing (Uddin et al., 2017), have not been found with significant results after multiple comparison corrections. Interestingly, the right vAI seed indicated the reduced rsFC with right medial SFG in results with GSR in children with PNE. Patients suffering from PNE are prone to various emotional problems and the above abnormal rsFC of vAI may play a role in this mechanism. Further studies need to be explored.

Another important result is that the right PI seed also showed decreased rsFC with the right medial SFG, while the left PI seed demonstrated decreased rsFC with the right SFG. The PI with connections to brain regions for sensorimotor processing is associated with receiving and integrating bodily signals and external stimuli (Uddin et al., 2017). In addition, the reduced cluster in the right medial SFG is largely located in BA10 (Table 2). BA10 is a part of mPFC and is mostly related to response inhibition, working memory, and cognitive flexibility (Lei et al., 2012b; Carlén, 2017). A resting-state fMRI study demonstrated that the brain local activity of BA10 was abnormal in children with PNE (Lei et al., 2012b). Yet, the left PI seed showed no significant clusters in results with GSR in the PNE group. Therefore, we should explain these inconsistent results cautiously and seriously.

On one hand, some studies expected that the GSR can introduce artifactual anticorrelations (Weissenbacher et al., 2009). On the other hand, it was found that the GSR can remove the effects of unrelated signals and improve analysis results (Liu et al., 2017). Only in the results with GSR, before and after setting age as a covariate, significantly and positively correlations between bladder volume and the rsFC of the left dAI with right medial SFG, and the rsFC of the right PI with right medial SFG were found in the PNE group. There was a stronger activity of the insula, especially the anterior insula at the full bladder condition compared to the lower bladder volumes in a task fMRI study (Kuhtz-Buschbeck et al., 2009). The prefrontal cortex, particularly the medial regions, is involved in bladder storage, and voiding function (Sakakibara et al., 1999). Children with PNE often have small functional bladder capacity. In a neonatal rat study, Ng et al. (2010) found that the central inhibition of spontaneous voiding was missing in neonatal rats after undergoing bladder volume reduction, suggesting that decreased neonatal bladder capacity might affect the brain–bladder control network. Moreover, abnormal brain function (e.g., dysconnectivity of the SN-DMN) may cause long-term alterations in bladder capacity or bladder fullness sensation and external urethral sphincter function, which could occur as incontinence in children with PNE. Another chronic intermittent hypoxia (CIH)-induced enuresis model in rats resulted in bladder instability and thereby induced enuresis by producing oxidative stress to promote overexcitation of sympathetic nerves (Su et al., 2020), suggesting the importance of brain–bladder dysfunction on enuresis. However, the inconsistent results without and with GSR in correlation analysis should be explained with caution. Further studies on the interplay between dysconnectivity of the SN-DMN and bladder dysfunction in animal models or humans are warranted.

## Clinical implications

Our neuroimaging study provides an opportunity to better understand the pathophysiology of PNE and, thus, look for potential targets for clinical intervention. Previous fMRI studies have also demonstrated a few abnormal brain areas associated with micturition control, arousal dysfunction, emotion processing, and cognitive ability (Lei et al., 2012b; Zhang et al., 2015, 2021; Wang et al., 2019). However, the exact mechanism of abnormal brain–bladder control networks in children with PNE is still a mystery. Functional brain imaging is considered promising not only to uncover the urological mechanisms, but also to develop novel and effective treatments in the future (Kitta et al., 2015).

## Limitations

There are some limitations to our study. First, our present study was cross-sectional and longitudinal studies showing changes in the brain after clinical interventions should be considered. Second, the relationship between brain function and behavior was not comprehensive and consistent in results without and with GSR and more useful clinical behavioral variables should be collected in the future. Last but not least, the number of subjects in the present study was relatively small and a larger longitudinal multimodel fMRI study should be considered.

## Conclusion

In summary, compared with HC subjects, PNE in children had the altered insular rsFC with the mPFC. Our study demonstrated the abnormal connection between the SN and the DMN in children with PNE, which may involve in the pathophysiology of children with PNE. These results contribute to a better understanding of the pathophysiology of PNE and the development of individualized treatment strategies.

## Data availability statement

The raw data supporting the conclusions of this article will be made available by the authors, without undue reservation.

## Ethics statement

The studies involving human participants were reviewed and approved by the Institutional Review Board (IRB) of Shanghai Children’s Medical Center, School of Medicine, Shanghai Jiao Tong University. Written informed consent to participate in this study was provided by the participants’ legal guardian/next of kin.

## Author contributions

SZ and JS completed all the data analysis relevant to this study and drafted the initial manuscript. MW and YM acquired the data. XD and JM revised the manuscript. All authors co-designed this study, reviewed and approved the final version of the manuscript.

## Conflict of interest

The authors declare that the research was conducted in the absence of any commercial or financial relationships that could be construed as a potential conflict of interest.

## Publisher’s note

All claims expressed in this article are solely those of the authors and do not necessarily represent those of their affiliated organizations, or those of the publisher, the editors and the reviewers. Any product that may be evaluated in this article, or claim that may be made by its manufacturer, is not guaranteed or endorsed by the publisher.

## Footnotes

1
http://www.fil.ion.ucl.ac.uk/spm/
