# Supplementary material for: Altered resting-state functional connectivity of insula in children with primary nocturnal enuresis
Source: Front Neurosci. 2022 Jul 19;16:913489. doi: 10.3389/fnins.2022.913489 (PMC9343997; doi:10.3389/fnins.2022.913489)
Supplement: Supplementary file 1 [file Data_Sheet_1.docx]

Supplementary Material

# Supplementary Tables

**Table S1** Brain regions showing significant differences in functional connectivity with insula or its subregions between PNE and HC children (with global signal regression).

| Seed | Brain region | Cluster size | MNI coordinates | | | Peak T value |
| --- | --- | --- | --- | --- | --- | --- |
|  |  |  | X | Y | Z |  |
| Left insula | Frontal_Sup_Medial_R (BA 10) | 480 | 6 | 51 | 6 | -4.1366 |
| Right insula | Frontal_Sup_Medial_R (BA 10) | 349 | 12 | 54 | 15 | -4.1101 |
| Right vAI | Frontal_Sup_Medial_R (BA 9) | 205 | 3 | 51 | 18 | -4.0778 |
| Left dAI | Frontal_Sup_Medial_R (NA) | 488 | 12 | 51 | 21 | -4.8796 |
| Right dAI | Frontal_Sup_Medial_R (BA 10) | 438 | 3 | 54 | 6 | -4.3595 |
| Right PI | Frontal_Sup_Medial_R (BA 10) | 283 | 12 | 51 | 3 | -4.0425 |

*PNE, primary nocturnal enuresis; HC, healthy control; MNI, Montreal Neurological Institute; BA, Brodmann areas; vAI, ventral anterior insula; dAI, dorsal anterior insula; PI, posterior insula; Frontal_Sup_Medial_R, right medial superior frontal gyrus.*

**Table S2** Correlations between clinical characteristics and FC z-values from significant brain regions in PNE subjects.

| Seed | Brain region | Clinical characteristic | r | P |
| --- | --- | --- | --- | --- |
| Left insula | Frontal_Sup_Medial_R (BA 9) | MDA | 0.188 | 0.296 |
|  |  | Frequency | 0.234 | 0.190 |
|  |  | BV | 0.224 | 0.209 |
| Right insula | Frontal_Sup_Medial_R (BA 9) | MDA | 0.120 | 0.506 |
|  |  | Frequency | 0.243 | 0.173 |
|  |  | BV | 0.143 | 0.427 |
| Left dAI | Frontal_Sup_R (NA) | MDA | 0.146 | 0.416 |
|  |  | Frequency | 0.330 | 0.060 |
|  |  | BV | 0.286 | 0.107 |
| Right dAI | Frontal_Sup_Medial_R (BA 9) | MDA | 0.093 | 0.608 |
|  |  | Frequency | 0.256 | 0.151 |
|  |  | BV | 0.172 | 0.337 |
| Left PI | Frontal_Sup_Medial_R (BA 10) | MDA | 0.176 | 0.328 |
|  |  | Frequency | 0.129 | 0.475 |
|  |  | BV | 0.274 | 0.122 |
| Right PI | Frontal_Sup_Medial_R (BA 10) | MDA | 0.096 | 0.593 |
|  |  | Frequency | 0.079 | 0.663 |
|  |  | BV | 0.294 | 0.097 |

*PNE, primary nocturnal enuresis; MDA, micturition desire-awakening; Frequency, bed-wetting frequency per week; BV, bladder volume; dAI, dorsal anterior insula; PI, posterior insula; Frontal_Sup_Medial_R, right medial frontal superior gyrus; Frontal_Sup_R, right superior frontal gyrus; BA, Brodmann areas; NA, not applicable.*

# Supplementary Figures


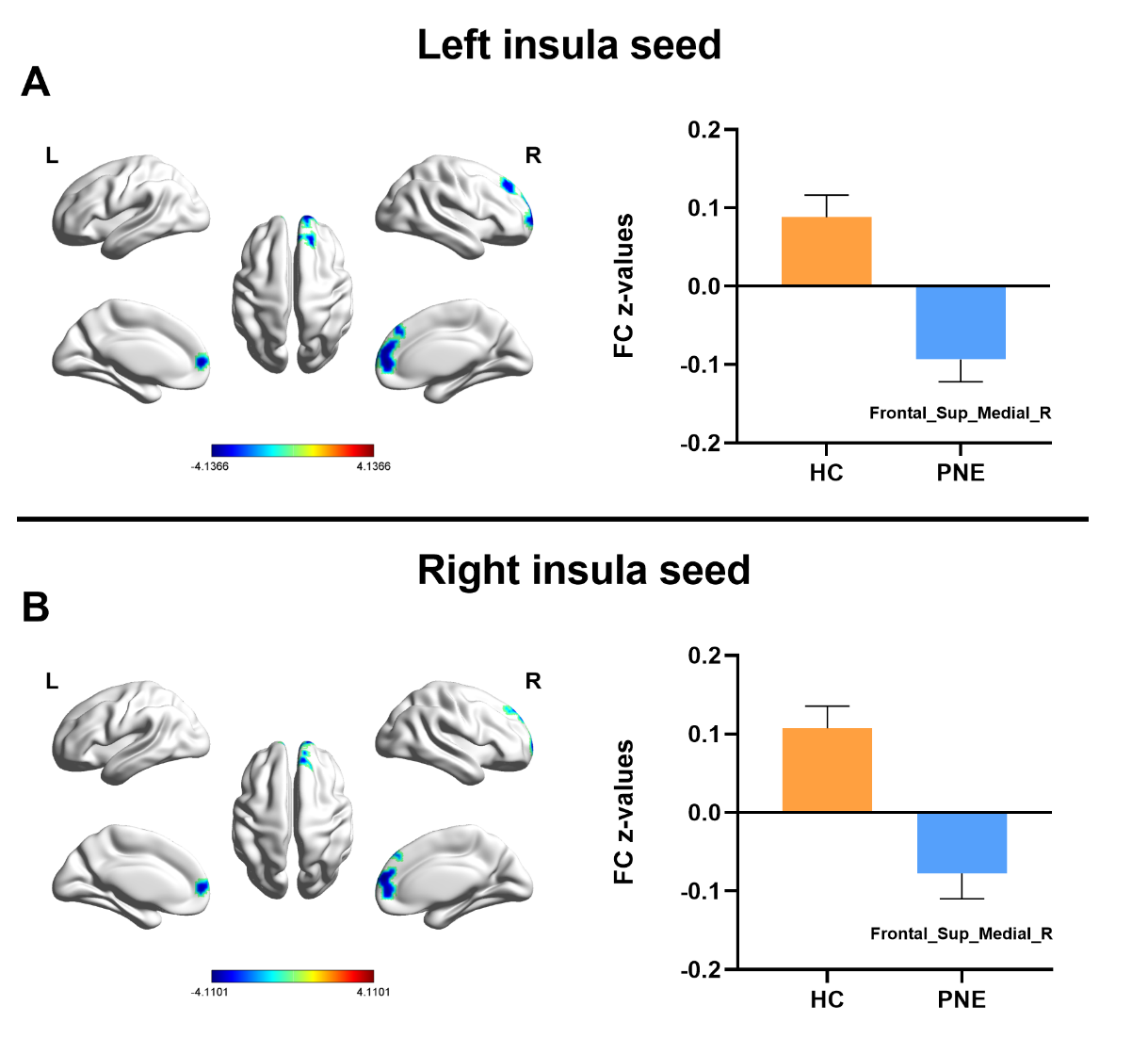


**Figure S1** Comparisons of the left and right insula-centered functional connectivity between PNE and HC children (with global signal regression). The (A) left and (B) right insula seeds indicating reduced functional connectivity with Frontal_Sup_Medial_R (two-tailed GRF correction, P < 0.01 at single voxel as well as P < 0.05 at cluster level). PNE, primary nocturnal enuresis; HC, healthy control; Frontal_Sup_Medial, medial superior frontal gyrus; L, left; R, right. Color scales: t value; Error bars: standard errors of mean.


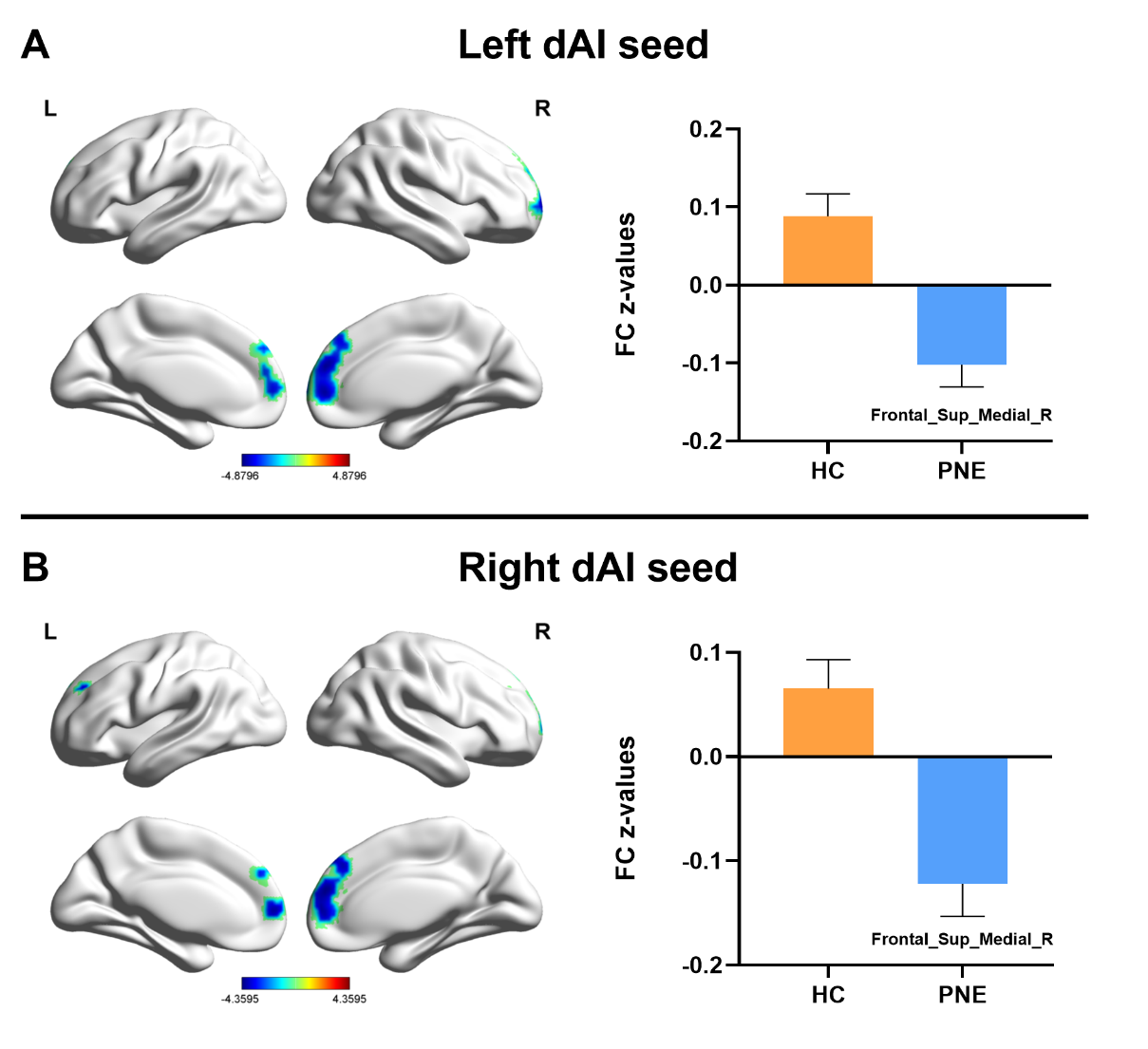


**Figure S2** Comparisons of the dorsal anterior insula-centered functional connectivity between PNE and HC children (with global signal regression). The (A) left and (B) right dAI seeds indicating reduced functional connectivity with Frontal_Sup_Medial_R (two-tailed GRF correction, P < 0.005 at single voxel as well as P < 0.05 at cluster level). PNE, primary nocturnal enuresis; HC, healthy control; dAI, dorsal anterior insula; Frontal_Sup_Medial, medial superior frontal gyrus; L, left; R, right. Color scales: t value; Error bars: standard errors of mean.


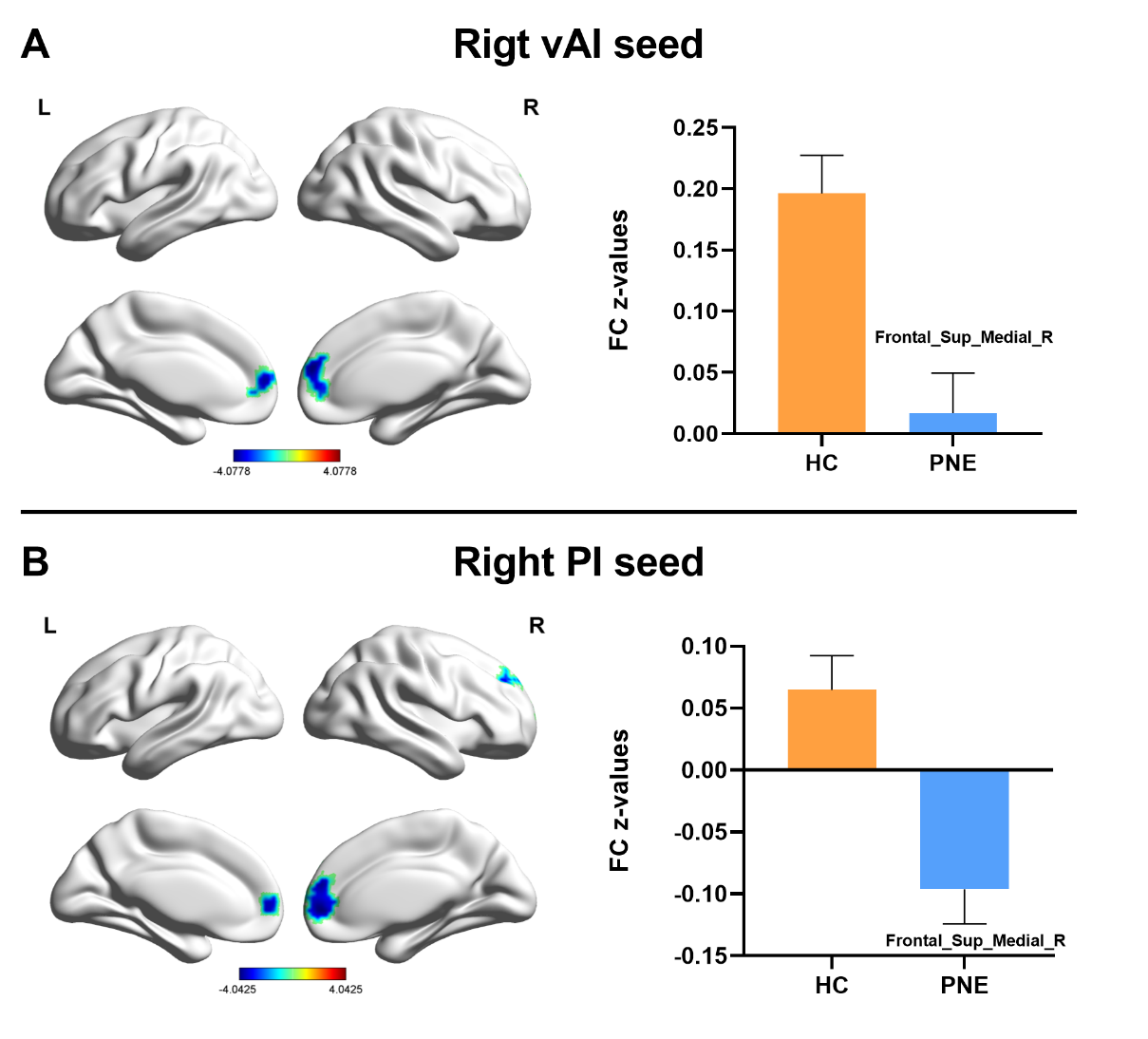


**Figure S3** Comparisons of the vAI-centered and PI-centered functional connectivity between PNE and HC children, respectively (with global signal regression). The (A) right vAI and (B) right PI seeds indicating reduced functional connectivity with Frontal_Sup_Medial_R (two-tailed GRF correction, P < 0.005 at single voxel as well as P < 0.05 at cluster level). PNE, primary nocturnal enuresis; HC, healthy control; vAI, ventral anterior insula; PI, posterior insula; Frontal_Sup_Medial, medial superior frontal gyrus; L, left; R, right. Color scales: t value; Error bars: standard errors of mean.
